# Distinct clinicopathological and genomic features in solid and basaloid adenoid cystic carcinoma of the breast

**DOI:** 10.1038/s41598-022-12583-w

**Published:** 2022-05-19

**Authors:** Juan Ji, Fang Zhang, Fanglei Duan, Hong Yang, Jun Hou, Yang Liu, Jie Dai, Qiong Liao, Xian Chen, Qingsong Liu

**Affiliations:** 1 Department of Pathology, Sichuan Cancer Hospital & Institute, Chengdu, 610041 Sichuan China; 2 Department of Pathology, Affiliated Hospital of Chengdu University of Traditional Chinese Medicine, Chengdu, 611137 Sichuan China; 3 Department of Pathology, Neijiang First People’s Hospital, Neijiang, 641099 Sichuan China

**Keywords:** Oncology, Breast cancer, Cancer genetics, Tumour biomarkers

## Abstract

Adenoid cystic carcinoma (AdCC) of the breast is a rare indolent carcinoma of salivary gland-type tumors, frequently associated with MYB genetic alteration. Solid and basaloid adenoid cystic carcinoma (SB-AdCC) is considered a sparse variant of AdCC. This study sought to search for clinicopathological and genomic features in SB-AdCC. Registered clinicopathological data on a cohort of 13 AdCC of the breast cases, including six conventional adenoid cystic carcinoma (C-AdCC) cases and seven SB-AdCC cases, were collected. MYB gene rearrangement via fluorescent in situ hybridization was investigated and MYB protein expression was evaluated by immunohistochemistry. Compared with C-AdCC, we found that the distribution of SB-AdCC cases were shifted to older age and were more frequently distant metastasis. Moreover, metastasis cases also showed a high (exceed 30%) Ki-67 index. Both groups showed MYB rearrangements and MYB protein expression, but they were less frequent in SB-AdCC than C-AdCC. To conclude, our results suggest that SB-AdCC is an aggressive variant of mammary AdCC with a higher incidence of distant metastases compared with C-AdCC, though they share common molecular features. A high Ki-67 index may be an adverse prognostic factor for metastasis.

## Introduction

Adenoid cystic carcinoma (AdCC) of the breast is a rare, peculiar subtype of triple negative breast cancer (TNBC), characterized by its excellent prognosis and very rare occurrence of metastases^1–5^. It is histologically similar to its counterpart in the salivary gland that presents a dual cellular composition (ductal and myoepithelial cell differentiation) and three major growth patterns: cribriform, tubular, and solid^6–9^. The molecular signatures of AdCC show MYB oncogene fusion with the NFIB transcription factor and MYB rearrangement. MYB–NFIB gene fusion, discovered in 2009, results in increased MYB protein expression that can be detected using immunohistochemistry^10–12^.

The solid and basaloid variant of breast adenoid cystic carcinoma (SB-AdCC) is currently perceived as an exceedingly rare histologic subgroup of AdCC, with only several published studies. It is classified into three grades by different solid components. Grade I tumors are those with tubular and cribriform areas but without solid components, whereas Grade II tumors are pure or mixed cribriform with less than 30% solid components, and tumors that have more than 30% solid components are classified as Grade III^13,14^. Owing to its rarity, there is a scarcity of data on the clinicopathological figure and repertoire of genetic alterations of this cancer.

This study aims to present the clinical features, pathological characteristics, and genetic repertoire of SB-AdCC compared with C-AdCC.

## Results

### Clinicopathological features

**Figure 1:**
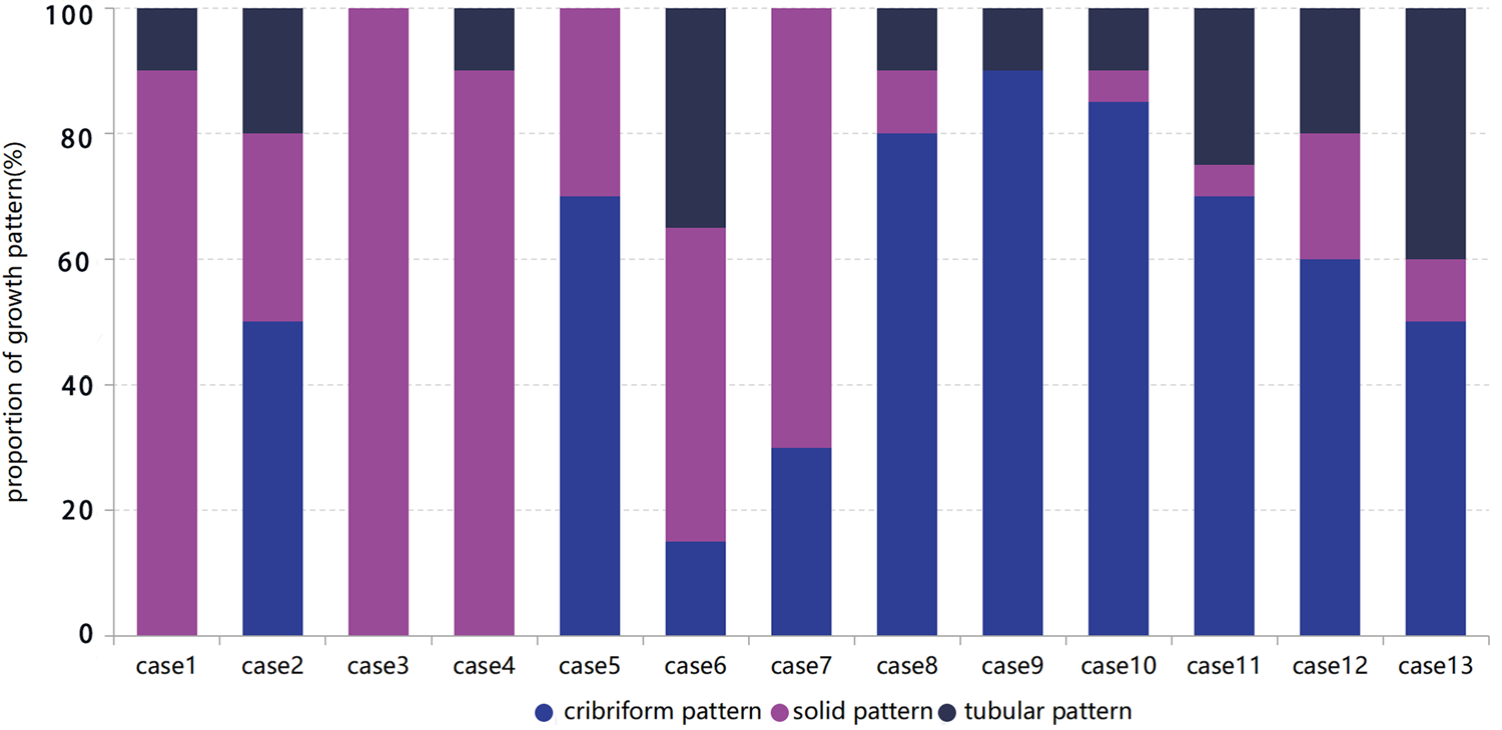
Histogram showing proportion of morphological growth pattern in AdCC patients. Case 1–7 is SB-AdCC group and Case 8–13 is C-AdCC group.

The clinicopathologic features of the study cohort consisting of 13 patients are summarized in Table 1. All cases were classified into two groups upon their three morphological patterns (Fig. 1). The first group was SB-AdCC (*n* = 7, 55%), which harbored a solid pattern of over 30%, as described in previous studies. The second group was C-AdCC (*n* = 6, 45%), with cribriform or a tubular and reticular pattern. All of these cases were female. The tumors that occurred in SB-AdCC were in older patients compared with C-AdCC (range 43–77, median 57 vs. range 37–51, median 44; *P* value < 0.05). The median tumor size of SB-AdCC was larger compared with C-AdCC (range 1.5–4, median 2.5 vs. range 1.1–2.3, median 1.7; *P* value > 0.05) but failed to achieve statistical significance.

**Table 1:** Clinicopathologic parameters of the breast AdCC cohort.

	*N* (%)
Total	13 (100)
**Age**	
≤ 50	9 (70)
> 50	4 (30)
**Tumor size (mm)**	
< 20	5 (38)
≥ 20	8 (62)
**Predominant growth pattern**	
Cribriform	7 (54)
Solid	5 (38)
Tubular	1 (8)
**Solid growth pattern**	
> 10%	1 (13)
> 30%	2 (25)
> 50%	3 (37)
> 70%	2 (25)
**Nottingham grade**	
1	3 (23)
2	8 (62)
3	2 (15)
**Final surgery**	
Lumpectomy	6 (46)
Mastectomy	7 (54)
**Chemotherapy**	
Yes	11 (85)
No	2 (15)
**Radiotherapy**	
Yes	2 (15)
No	11 (85)
**Lymph node status**	
Negative	13 (100)
Positive	0 (0)
**Metastasis**	
Yes	2 (15)
No	11 (85)
**Perineural invasion**	
Yes	5 (38)
No	8 (62)
Ki-67 index	
≤ 30%	11 (85)
> 30%	2 (15)

**Figure 2:**
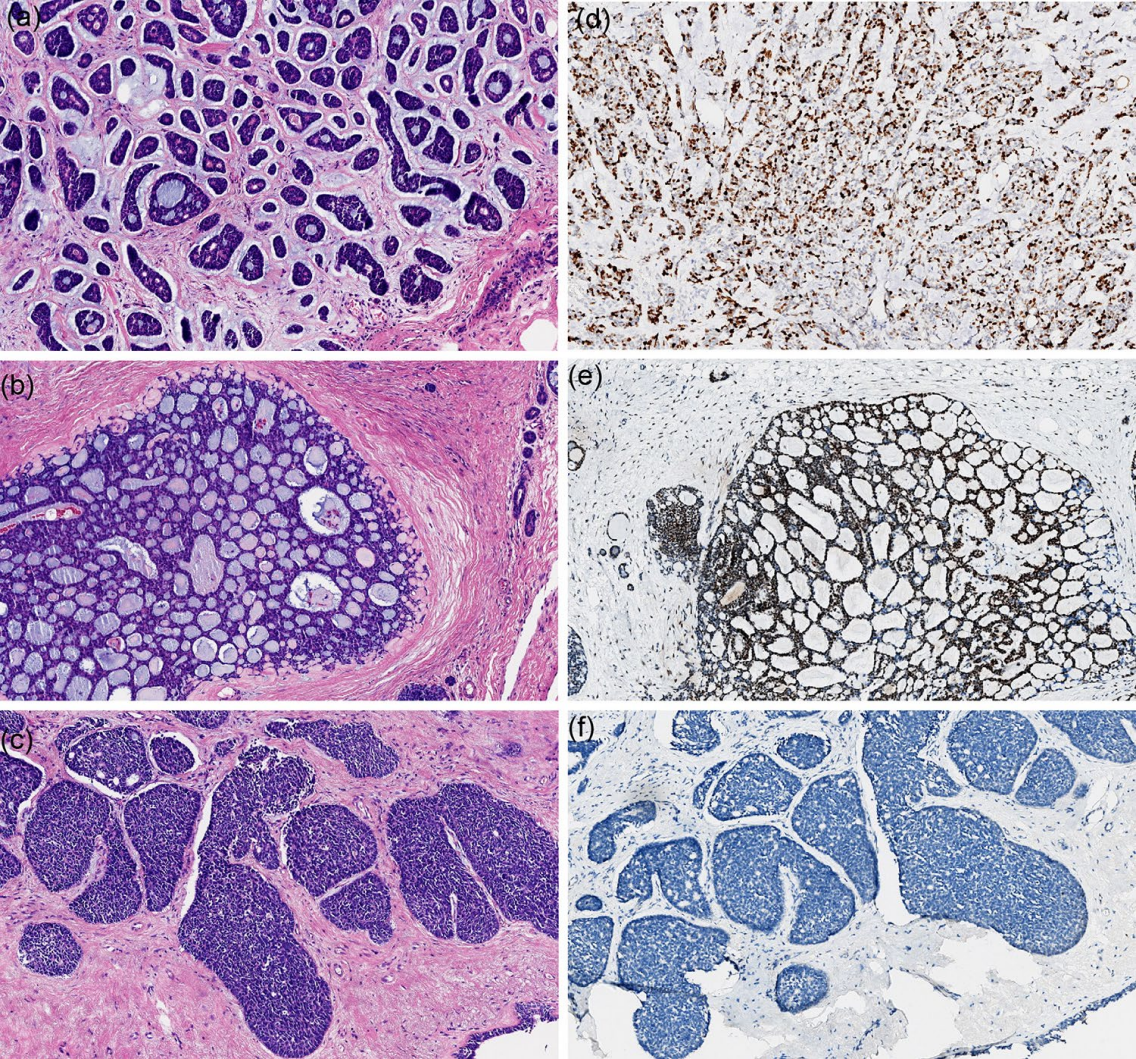
Morphological Features of Classic and Solid-basaloid Breast AdCCs. (**a**–**c**) exhibit three histological characteristics of hematoxylin and eosin (H&E) images in AdCC; (**a**) AdCC with cribriform and tubular growth; (**b**) conventional cribriform growth with accumulation of basal membrane material in pseudoluminae; (**c**) showing solid-basaloid growth pattern; (**d**) presents a high Ki-67 index of immunostaining; (**e**,**f**) represent micrographs of MYB expression (strong and diffuse positive and negative, respectively).

Conventional AdCC showed permutation and combination of cribriform and tubular or reticular patterns (Fig. 2a,b). Both true and false glandular lumens were easily recognized. True glandular lumens are usually small and contain eosinophilic/basophilic secretions. The tumor nuclei often present low to intermediated grades. According to the Nottingham grade system, all the nuclear grades of C-AdCC were low grade (four cases were Grade 1 and two cases were Grade 2). For molecular typing, apart from one case, PR immune staining was positive; all the C-AdCC cases in our study were TNBC. The proliferative index Ki-67 for all C-AdCC cases were less than 20%.

SB-AdCC is mainly composed of solid tumor nests of cells with a myxoid or hyalinized and desmoplastic stroma (Fig. 2c). Compared with C-AdCC, it is tough to recognize the true glandular lumens. The cell cytoplasm in the true glandular lumen is more abundant and eosinophilic. Some tumor cells surrounding the nests are arranged in a palisade pattern. The cytoplasm of the cells is sparse, with medium to heavy atypia, round or oval nuclei, uniform chromatin, and unclear nucleoli. All SB-AdCC cases were TNBC (7/7, 100%). Most of this group had a low Ki-67 index, but two cases with metastasis had a high Ki-67 index (above 30%; Fig. 2d).

### Assessment of MYB status in IHC and FISH rearrangement

**Figure 3:**
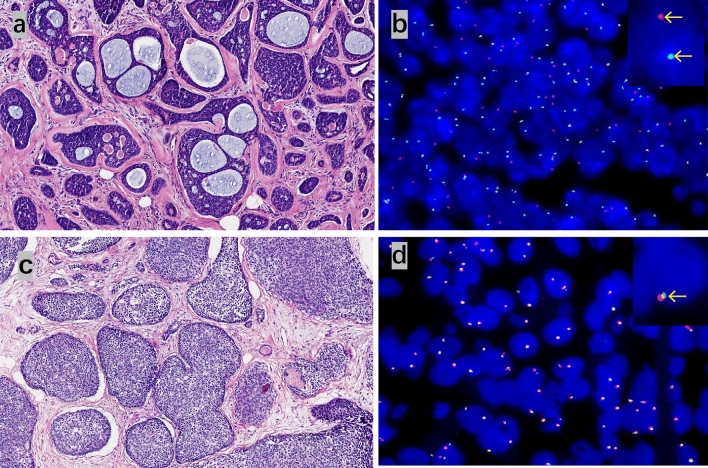
The MYB fluorescence in situ hybridization in classic and solid-basaloid AdCCs. Envoy H&E micrograph and corresponding FISH image using MYB break-apart assay demonstrating red and green signals for separation of C-AdCC in (**a**) and (**b**); (**c**) and (**d**) represent H&E and FISH micrographs in SB-AdCC with no rearrangement involving MYB.

We detected MYB status with immunochemistry and FISH arrangement in all cases, considering that MYB in immune staining is more sensitive than that in FISH arrangement^11^. Overall, 8 of 13 cases (60%) showed MYB protein expression (Fig. 2e,f), and 7 of 13 cases (54%) showed MYB rearrangement by FISH (Fig. 3). Most conventional AdCC cases had a positivity for MYB and rearrangement. Only one case loss the expression of MYB protein while being negative for rearrangement, whereas MYB protein expression and MYB rearrangements were presented in 4 of 7 (57%) tumors classified as SB-AdCC. In the seven SB-AdCC cases, three had positivity for MYB immune staining and rearrangements in common, and two cases had the same negative for MYB protein expression and rearrangements. The other two cases both were positive for MYB rearrangements but negative for MYB immunohistochemical staining. In our analyses, MYB protein labeling may not have been as sensitive as the MYB rearrangements associated with the aforementioned studies^11,15^.

### Treatment and outcome

**Figure 4:**
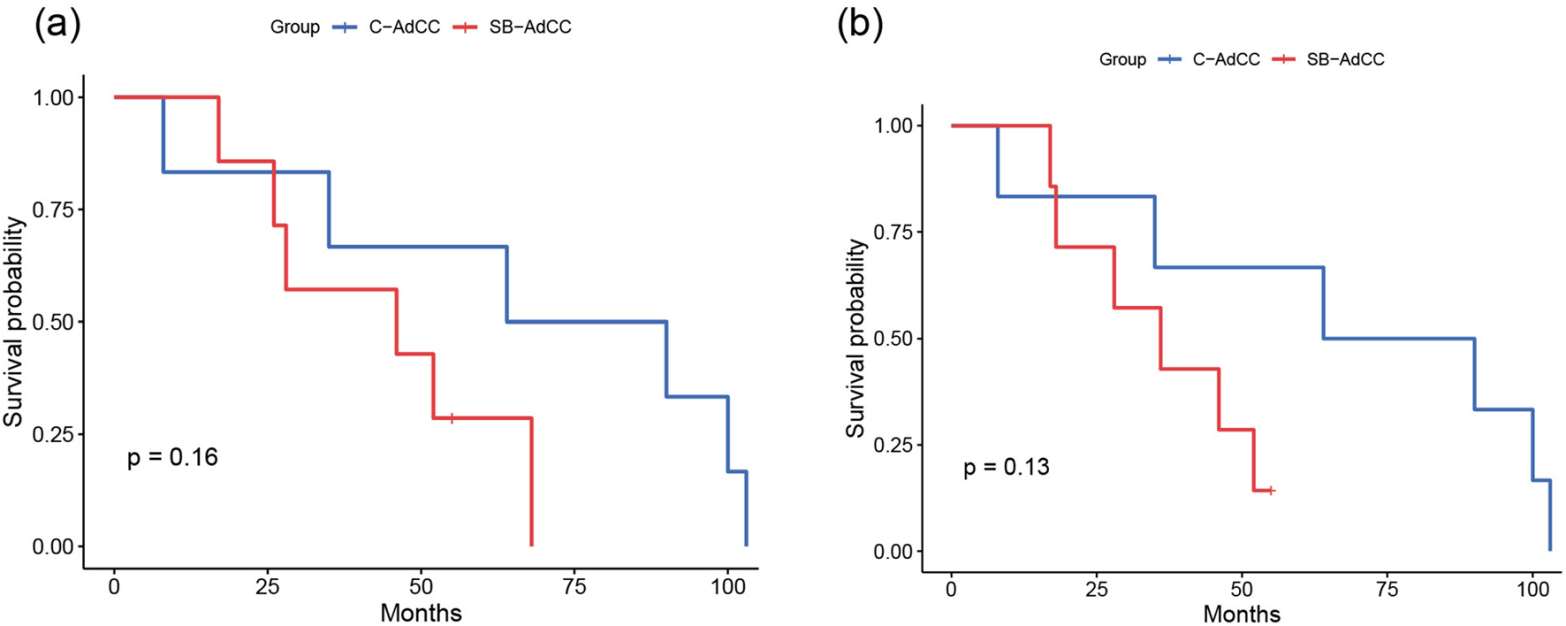
Overall survival (**a**) and disease-free survival analysis (**b**) in classic and solid-basaloid breast AdCCs.

Owing to the scarcity of AdCC in breast, there is scarce data on its clinical treatment. In our study, all cases received surgical treatment. In the C-AdCC group, two patients underwent an operation for lumpectomy and four patients underwent mastectomy. No patients with C-AdCC received adjuvant radiation, but all cases (6/6, 100%) received adjuvant chemotherapy. The initial surgical treatment for the SB-AdCC group was five lumpectomies and two mastectomies. Five patients underwent adjuvant chemotherapy and two patients underwent adjuvant radiation**.** All cases were followed up for 17–103 months, with a mean time of 57 months (Table 2). All (6/6) patients with conventional AdCC were disease-free by a mean time of 67 months. The overall survival and disease-free survival of SB-AdCC showed lower numbers (42 months, 36 months) compared with C-AdCC (67 months, 67 months), although it did not reach statistical significance (Fig. 4). No patients had lymph node metastasis in our study. Two cases of SB-AdCC had distant widespread, and one case died from another disease. One patient (Case 2) had a lung metastasis after 2.5 years with a lumpectomy and adjuvant chemotherapy. Following radiation treatment, the patient kept a stable condition. The other patient (Case 5) had a liver metastasis after 1.5 years with a mastectomy and adjuvant chemotherapy. They did not receive radiation because of the intolerance of their body, but they were alive with AdCC after 26 months. The tumor in Case 2 was positive for both MYB protein expression and rearrangement. Conversely, Case 5 was negative for both MYB immune staining and rearrangement. Notably, two cases of the distant metastasis had a high Ki-67 index (40% and 50%).

**Table 2:** Treatment and outcome of the SB-AdCC and C-AdCC Groups.

Number	Subtype	Age, sex	Tumor size (mm)	LN status	Treatment	Distant metastasis	DFS	OS	Clinical status
Case1	SB-AdCC	77, F	35	0/4	M	No	55	55	DOC
Case 2	SB-AdCC	50, F	15	0/5	L, CT, RT	Yes (lung)	36	68	AWD
Case 3	SB-AdCC	48, F	30	0/16	L, CT	No	52	52	NED
Case 4	SB-AdCC	43, F	20	0/13	L, CT, RT	No	46	46	NED
Case 5	SB-AdCC	59, F	40	0/2	M, CT	Yes (liver)	18	26	AWD
Case 6	SB-AdCC	64, F	19	0/7	L, CT	No	28	28	NED
Case 7	SB-AdCC	60, F	15	0/2	L	No	17	17	NED
Case 8	C-AdCC	42, F	23	0/4	L, CT	No	35	35	NED
Case 9	C-AdCC	51, F	11	0/11	M, CT	No	8	8	NED
Case 10	C-AdCC	41, F	12	0/17	L, CT	No	90	90	NED
Case 11	C-AdCC	49, F	20	0/14	M, CT	No	64	64	NED
Case 12	C-AdCC	37, F	20	0/21	M, CT	No	100	100	NED
Case 13	C-AdCC	44, F	15	0/17	M, CT	No	103	103	NED

*F* female, *L* lumpectomy, *M* mastectomy, *CT* chemotherapy, *RT* radiotherapy, *DOC* death of other cause, *AWD* alive with disease, *NED* no evidence of disease.

## Discussion

AdCC of the breast is a scarce, distinctive salivary gland-type malignant tumor that accounts for 0.1%–3% of all breast tumors^6,16,17^. It is an aggressive neoplasm in the salivary gland but its counterpart in breasts has a favorable clinical course and few cases with lymph nodes and distant metastases, especially compared with other TNBC^3,15^. After Patey and Thackray first showed that the solid growth pattern is bound up with adverse prognosis of AdCC^18^, a handful of groups have further researched SB-AdCC. To shed additional light on this disease, the current study characterizes the clinicopathological and molecular figures in the Sichuan Cancer Hospital & Institute.

MYB proteins express in AdCC at different sites, driven by MYB gene amplification, MYB or MYBL1 rearrangements, or MYB-NIFI fusion genes^12^. After Persson identified this molecular alteration in AdCC, more researchers have shown that MYB is a specific molecular change at a different frequency^19^. In salivary gland tumors, about one-third to two-thirds of cases depicted MYB gene alteration^20–22^, which is represented in breast AdCC approximately 38–100% of the time^3,19^. The tumors showed no SB-AdCC features such as a high grade or atypical nucleus in the aforementioned studies. In our study, we explored two morphologic groups of AdCC. One group was conventional AdCC with low- or medium-grade nuclear features. The other group was high-grade AdCC with a solid and basaloid growth pattern (SB-AdCC). MYB protein expression of immunostaining and MYB rearrangement were 60% and 54%, respectively, which was higher than the previously scattered reports^14,16^. Despite this, akin to the study by Schwartz et al., both MYB protein expression and MYB rearrangement in the SB-AdCC group were lower than that of C-AdCC^23^.

Most AdCC cases showed a high degree of agreement in MYB protein and gene level results, except for two cases (Case 4 and Case 7) in the SB-AdCC group. Both cases showed positive MYB rearrangement and negative MYB proteins. The same results were obtained after repeated immune staining and FISH rearrangement under the same conditions. Of note, this is not an isolated finding. Different tissue types may differ in their sensitivity to protein expression, as prior studies have shown^21,24^. Thus, we surmise that one of the possible reasons was that it took too long for our formalin-embedded tissue to result in decreasing sensitivity of MYB protein expression. Previous studies on the transcriptional activity of MYB also suggest that diversity of the alternative splice RNA form of MYB or the break point of MYB rearrangement may produce proteins with different quantitative and qualitative activities^25,26^.

In addition to AdCC, the expression of MYB may be expressed in other tumors, such as pleomorphic adenoma, adenomyoepithelioma, and some TNBCs^3,15,27^. However, without MYB expression or MYB genomic rearrangement, diagnosis of AdCC could not always be ruled out, as presented in our study and prior research^11,23,28^. Therefore, we need to combine morphological and other immunomarkers to identify these tumors. Morphologically, adenomyoepithelioma and pleomorphic adenoma have a nodule and clear-border growth pattern, with a papillary or tubular structure. AdCC has conventional cribriform and a tubular, solid, and infiltrating growth pattern. Loss of myoepithelium is the key distinguishing feature between TNBC and AdCC; some immunomarkers such as P63 or calponin could aid recognition. When poor differentiation of SB-AdCC is encountered, it is tough to discern this disease from others; but this could be aided by looking for the classical histopathological section in tumor and MYB tests.

Compared with C-AdCC, SB-AdCC is more responsive to its clinical progress. In this study, there was no case with lymph node involvement, as there has been in previous studies^29,30^. However, two cases with lung and liver metastasis were presented, which is consistent with the findings of Slodkowska et al.^8^. In these two visceral metastasis cases, positive expression of MYB was found in both breast and primary lung metastases. However, the patient with liver metastasis was negative for MYB protein and gene rearrangement in both the primary and metastatic sites. This also supports previous studies, where MYB changes may not be associated with the prognosis^20,23^.

Interestingly, unlike what has been reported in the previous literature, we were the first to report AdCCs with highly proliferative activity, and this finding was reinforced in the two metastasis cases of this study. In the two metastatic patients, Ki-67 expression was high in both the primary and metastatic sites. As is well known, Ki-67 is a useful and predictive clinical indicator for breast cancer subtype and pathological responses of neoadjuvant endocrine therapy. A great deal of analyses and research indicates that breast cancer patients with a high Ki-67 index have a higher risk of recurrence and a worse survival rate^31,32^, which may be germane to AdCC. This suggests that treatment of AdCC with high Ki-67 expression may require more therapeutic methods, including but not limited to segmentectomy, radiotherapy, and chemotherapy. With a finite dataset in this study, further studies are needed.

Because of the relative rarity of AdCC in breasts, there is still no consensus on how to treat this disease. With the rapid application of radiotherapy technology, lumpectomy with radiation treatment has been one of the better choices for AdCC patients^33,34^. In a study by Khanfir, the locoregional control rate was significantly different between the radiotherapy group and the group that underwent surgery alone^35^. A recent study by Gomez-Seoane et al. used SEER data (2005–2015) to evaluate the benefit of radiotherapy in patients with breast AdCC. They retrospectively studied 488 patients and found the overall survival rate of 244 cases with postoperative radiation was better than that of the control group, denoting radiation needs to be considered after surgery for patients with breast AdCC^36^. Our study showed a higher proportion of mastectomy (6/13.46%), which may be the reason for the low proportion of radiation therapy. Currently, the optimal adjuvant chemotherapy for AdCC is not clear. Prior studies have shown a limited role for chemotherapy in AdCC^37^. Therefore, the choices of different doctors have been mixed. In our study, all patients underwent segmentectomy and most patients received chemotherapy (11/13, 85%), including the two metastasis cases, which appears to be more active than prior studies. About 80% cases received adriamycin, cyclophosphamide, and albumin-bound taxol. The liver metastasis patient (Case 5) with SB-AdCC received neoadjuvant therapy and surgery treatment in accordance with NCCN guidelines^38^. After the operation, carboplatin and capecitabine sequential therapy were used. All cases were kept stable, with a follow-up of 26–68 months. Despite the high frequency of chemotherapy treatment in our study, there was a limited, small sample in our data, which still did not show a difference in outcome between the chemotherapy and non-chemotherapy groups. Therefore, more studies are needed to explore treatment.

By virtue of the low incidence rate, the sample size of this study is still small. In addition, previous studies have shown that MYB protein can be expressed not only in AdCC, but also in other salivary gland tumors. Some of the literature has suggested that the two transformation mechanisms may be different^23^. In AdCC, MYB is expressed by fusion with NFBI, whereas in other tumors MYB amplification may be achieved. More data on this needs to be studied and discussed.

In summary, SB-AdCC is an aggressive variant of mammary ACC with a higher incidence of distant metastases compared with C-AdCC, but they share common molecular features. A high Ki-67 index may be an adverse prognostic factor for metastasis.

## Methods

### Patient section

This retrospective analysis included 13 cases of conventional and solid-basaloid variant breast AdCCs diagnosed at the Department of Pathology, Sichuan Cancer Hospital & Institute between 2013 and 2021.The clinical parameters of the AdCCs, including age, sex, year, tumor size, treatment, lymph node status and metastases, and clinical outcome were retrieved when available. We also reviewed each case with pathological/histological features according to the standards proposed by the World Health Organization in 2019^39^. As previously described, SB-AdCCs were defined as having a solid-basaloid growth pattern in at least 30% of the tumor area, which was also referred to as a high grade of AdCC.

### Immunohistochemistry

Unstained sections were prepared for MYB, CK5/6, p63, CD117, estrogen receptor (ER), progesterone receptor (PR), HER2, and Ki-67 immunohistochemistry. The MYB immune staining platform was Ventana Benchmark (Roche), following antigen retrieval with a cell conditioning solution (CC1; Roche). Tissue sections were incubated with a rabbit monoclonal antibody to recognize the N-terminus of c-MYB (Abcam ab45150, clone EP769Y at 1:200 dilution and detected by the OptiView DAB detection system; Roche). The following Ventana-prediluted antibodies were used: cytokeratin 7, cytokeratin 5/6, p63, CD117, ER, PR, HER-2, and Ki-67. All cases were reviewed independently by two pathologists (QL and FLD). Over 10% nucleus expression of MYB was defined as positive, as previously described. ER, PR, and HER2 expression by immunohistochemical stains were assessed according to current American Society of Clinical Oncology/College of American Pathologists (ASCO/CAP) guidelines^40^. The Ki-67 index was assessed according to international recommendations on the assessment of Ki-67 in breast cancer^41^. Nuclear expression in at least 10% of cells was scored as positive for P63 and cytoplasm-scattered cancer cells were scored as C-kit positive, as previously reported^7^.

### Fluorescence in situ hybridization of MYB

MYB gene rearrangements were detected on 4-μm-thick paraffin-embedded sections from selected blocks by an ampipine MYB (6q23) gene fracture probe reagent according to the manufacturer’s instructions. A dual-color break-apart interphase FISH assay was performed using centromeric (BAC clone RP11-349 J5; red) and telomeric (BAC clone RP11-641019; green) probes. Probe labeling, hybridization, post-hybridization washing, and fluorescence detection were performed according to procedures established at the Molecular Cytogenetics Core Facility. Slides were scanned using a Leica Dm6000 epifluorescence microscope equipped with cytology imaging software.

If red and green signals were separated by more than two hybridization signals (split-apart signals), or if there were extra single red or green signals in more than 30% of the nuclei in a case, we calculated them as positive. At least 100 nuclei per case were evaluated.

### Statistical analysis

Fisher’s exact test and the Mann–Whitney U test were used for comparisons between categorical variables and continuous variables, respectively. All statistical analyses were performed using SPSS 21.0. Distant disease-free survival and overall survival were calculated from the date of diagnosis to the date of any recurrence or distant recurrence, respectively.

### Ethical approval

Informed consent was obtained in all cases, and the study was approved by the Ethics Committee of Sichuan Cancer Hospital & Institute (SCCHEC-02-2022-011). All analyses were performed in accordance with the relevant guidelines and regulations.

## Data Availability

The datasets generated and/or analyzed during the current study are available from the corresponding author on reasonable request.
